# En-risk to de-risk: An iterative human factors framework for scale development in emerging technologies

**DOI:** 10.1016/j.jdin.2025.12.001

**Published:** 2025-12-04

**Authors:** Jonathan Kantor, Michael Morrison, Samantha Vanderslott, Robert C. Carlisle

**Affiliations:** a Oxford Vaccine Group, University of Oxford, Oxford, United Kingdom; b Department of Engineering Science, Institute of Biomedical Engineering, University of Oxford, Oxford, United Kingdom; c Centre for Health, Law, and Emerging Technologies (HeLEX), University of Oxford, Oxford, United Kingdom

**Keywords:** biotechnology innovation, de-risking, human factors research, human-technology interaction, minimum desirable product (MDP), minimum viable product (MVP), product–market fit, psychometric scale development, risk assessment and derisking, technology adoption, transcutaneous vaccine delivery, vaccine hesitancy

Developing and implementing novel biotechnology solutions requires alignment between research and development (R&D) teams, investors/funding agencies, end users, and the public at large.^1^ Early-stage ventures traditionally focus on R&D and the race for a minimum viable product (MVP), shunting concern regarding human factors research—including an exploration of public preferences, use cases, demand, valuation, and likely adoption—downstream in the product development cycle. In this commentary, we outline a minimum desirable product (MDP) framework that brings validated human factors scales into the earliest phases of biotechnology R&D.

A significant proportion of biotechnology ventures fail due to lack of end-user buy-in or funding limitations, rather than technical or regulatory challenges,^2^ so that creating desirable products with a clear value proposition and viable odds of achieving product-market fit is critical even at the earliest stages of development. Shifting human factors work earlier in the process and approaching it as an integral part of the R&D framework, rather than as an afterthought, and developing scales to quantify and understand otherwise unquantified human factors, may therefore help reduce late-stage attrition rates.^3^

This approach may allow nascent biotechnology ventures to leapfrog existing approaches with higher technology readiness levels by accelerating the creation of a product that is not only viable but desirable as well (Fig 1). By satisfying quantitatively assessed thresholds for end-user and public acceptance, research groups can move from viability to desirability and from the MVP to the MDP.

**Fig 1:**
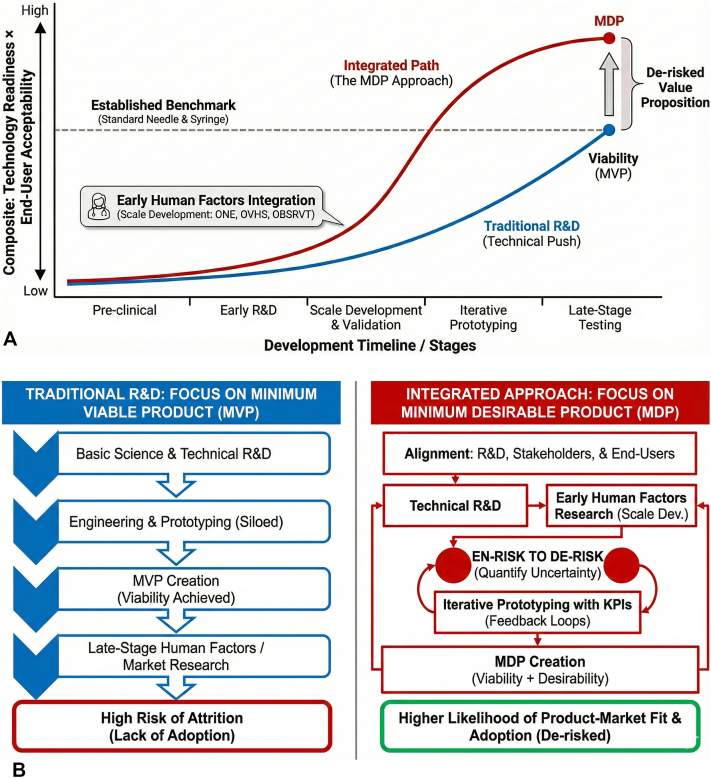
**A,** The synergy between traditional research and development *(*R&D*)* and human factors research demonstrates the potential to leapfrog existing technologies by applying minimum desirable product *(*MDP*)* principles. Conceptual comparison of traditional R&D (technical push) focused on achieving a minimum viable product (MVP) versus an integrated MDP approach that incorporates early human factors research and validated scale development. Early scale development and iterative use can convert uncertainty into measurable risk, enabling leapfrogging beyond existing benchmarks (eg*,* standard needle-syringe vaccination) and a more clearly de-risked value proposition. **B,** Traditional R&D focuses on the MVP, while incorporating human factors research early allows for a focus on the MDP. Schematic comparison of a traditional R&D pathway focused on achieving an MVP versus an integrated pathway aimed at an MDP. In the MVP approach, human factors and market research are deferred to late stages, increasing the risk of attrition and nonadoption. In the MDP approach, early human factors research, scale development, and key performance indicator (KPI)-driven feedback loops are integrated with technical R&D, converting uncertainty into measurable risk (en-risk to de-risk) and increasing the likelihood of product-market fit and adoption.

## From uncertainty to risk: en-risk to de-risk

The need to quantify key outcomes in R&D is a given; yet in human factors research, qualitative intuition often predominates, and the mere assertion that such research was conducted is sometimes seen—like a disclosure of conflicts of interest—to be all that is needed. Instead, to make de-risking possible, quantifying human factors uncertainty can convert uncontrollable unknown unknowns into known unknowns: quantifiable, manageable risks. We use “en-risking” to describe the deliberate quantification of otherwise unmeasurable stakeholder uncertainty so that it can be managed like any other R&D risk. To de-risk biotechnology, nebulous uncertainty must first be converted into measurable risk: en-risk to de-risk.

## Instrument-based risk quantification

Developing and validating novel instruments and scales is a replicable approach to rigorously quantify patient and public preferences across diverse technology classes and stakeholder groups. Using such instruments can reduce noise when studying public sentiment while simultaneously developing quantitative key performance indicators to track enthusiasm for product cycles, compare nascent and existing technologies, enhance narrative storytelling, and ultimately permit product de-risking.^4^^,^^5^ Since scales can be reused across studies and technologies, they effectively become shared infrastructure for de-risking innovation at the portfolio or ecosystem level. This applies to drug-device combinations, novel diagnostics, and, increasingly, to AI-enabled clinical tools where clinician and public acceptance remains poorly quantified. Moreover, these instruments permit the detection of the often nonlinear relationships between attitudes and behaviors, including threshold effects where small changes in sentiment could precipitate disproportionate shifts in adoption--information that would not be obvious from qualitative assessments alone.

Beyond the putative product development benefits, developing validated scales represents a prosocial contribution that yields outsized positive externalities, whether for academic institutions, start-ups, or established companies. Indeed, there is a virtuous feedback loop where instruments developed to accelerate internal product iteration can be tapped as evidence of viability for funding agencies and even repurposed by other academic and commercial users, allowing for stronger funding applications while attracting partners, visibility, and financial support.^4^

## Case example: transcutaneous ultrasound-mediated vaccine delivery

Nowhere is the urgent need for better understanding end user and public sentiment clearer than in vaccine development. In developing a transcutaneous ultrasound drug-device combination, making the narrative case for our technology required quantification of several key areas: needle fear, vaccine hesitancy, and preference for a nascent technology over needle and syringe. While some scales for the first 2 existed, none met a priori thresholds for multidimensionality and psychometric robustness. Three new instruments were therefore developed and validated on demographically representative populations with more than 3000 participants in the United Kingdom and in the United States: the Oxford Needle Experience scale,^6^ the Oxford Vaccine Hesitancy Scale,^7^ and the Oxford Benchmark Scale for Rating Vaccine Technologies.^8^

This approach produced a reusable toolkit for the rapid, iterative, and quantitative comparison of any vaccine-delivery technology to standard needle and syringe benchmarks, while also revealing a preference for ultrasound mediated vaccination, particularly among those who are most vaccine hesitant.^9^ By developing broad scales, investigators can now compare outcomes (using the Oxford Benchmark Scale for Rating Vaccine Technologies) across a range of subpopulations with risk factors for adoption enthusiasm (using the Oxford Needle Experience scale and the Oxford Vaccine Hesitancy Scale). Subscales provide even greater granular detail to explore what aspects of a product are most associated with potential adoption, allowing for rapid prototype iteration while making a stronger narrative argument to funding agencies and the public. Critically, this methodology is technology agnostic and transferable to any emerging area where public or user acceptance is uncertain.

## From the MVP to the MDP

Just as basic science and clinical data drive product development forward in an open, creative fashion,^10^ a parallel system of early-stage human factors research can be pursued to both increase funding opportunities and encourage adoption and venture success. Broadening the definition of target validation beyond the biological targets of drugs and devices to include stakeholder readiness can aid in developing the MDP.^2^

Quantification may also help mitigate the McNamara fallacy, where unquantified uncertainty is often ignored, though it may concomitantly introduce the risk of triggering Goodhart’s law—the principle that once a measure becomes a target, it ceases to be a reliable measure. The benefits of quantification are thus particularly profound when instruments are designed a priori to maximize construct validity, rather than treated as optimizable targets. In complex adaptive systems--whether biological, sociotechnical, or algorithmic--the act of measurement itself may also generate second-order effects that require ongoing calibration. Still, by (1) en-risking and de-risking through validated scale development, (2) deploying those scales repeatedly across prototype iterations to track shifts in stakeholder readiness, and (3) treating quantitative human factors data as key performance indicators for both product development and narrative storytelling, research groups and firms can pivot from an MVP to an MDP—potentially speeding development, improving adoption, and increasing the chances of venture success. As biotechnology increasingly intersects with artificial intelligence and algorithmic decision-making under uncertainty, human factors frameworks become even more critical for understanding adoption dynamics and second- or third-order effects, particularly when factors such as trust, perceived risk, or behavioral thresholds determine uptake.

## Conflicts of interest

None disclosed.
